# Mechanotherapeutic Modulation of the Nasal Microenvironment: RAMPA-Induced Maxillofacial Remodeling and Its Pathophysiological Impact on Mucus Rheology and Chronic Inflammation

**DOI:** 10.3390/bioengineering13060648

**Published:** 2026-05-30

**Authors:** Yasushi Mitani, Yuko Okai-Kojima, Mohammad Moshfeghi, Bumkyoo Choi, Yoshiya Hashimoto

**Affiliations:** 1 Codomo Clinic, Tokyo 180-0004, Japan; mitani@trust.ocn.ne.jp; 2 Private Practice, Children and Women Dental Clinic, Tokyo 106-0046, Japan; yukoyukono@gmail.com; 3 Independent Researcher, 7 Broadway Mansions, Coventry CV56NR, UK; mohammad.moshfeghi@gmail.com; 4 Department of Mechanical Engineering, Sogang University, Seoul 04107, Republic of Korea; 5 Department of Biomaterial, Osaka Dental University, Hirakata 573-1121, Japan; yoshiya@cc.osaka-dent.ac.jp

**Keywords:** RAMPA, mechanotransduction, Bone Morphogenetic Protein-2 (BMP-2), chronic sinonasal inflammation, shear-thinning mucus rheology, sinonasal homeostasis

## Abstract

Background: Pediatric maxillary deficiency often leads to upper airway constriction and chronic sinonasal inflammation. While conventional expansion focuses on dental width, the Right Angle Maxillary Protraction Appliance (RAMPA) system targets three-dimensional skeletal remodeling. This study investigates the mechanotherapeutic impact of RAMPA on the nasal microenvironment, specifically focusing on bone remodeling triggers and mucus rheology. Methods: Pre- and post-treatment CBCT data from 20 pediatric patients were analyzed. Computational Fluid Dynamics (CFD) and Finite Element Analysis (FEA) were employed to evaluate mechanical strain patterns and aerodynamic changes. We specifically identified the “BMP-2 TRIGGER ZONE” where tensile stress induces osteogenic signaling. Mucus clearance efficiency was modeled using the Carreau–Yasuda rheological framework. Results: RAMPA treatment resulted in a 61.2% mean increase in sinonasal volume (*p* < 0.0001), significantly outperforming natural growth baselines. FEA revealed that anterosuperior force vectors concentrated tensile stress on circummaxillary sutures, reaching thresholds for BMP-2 upregulation. CFD simulations demonstrated a significant reduction in wall shear stress (WSS) and improved airflow distribution, facilitating the transition of mucus from a high-viscosity state to a fluid state via shear-thinning effects. Conclusions: Our findings suggest that RAMPA-induced remodeling acts as a mechanotherapeutic modulator. As a proof-of-concept study, by triggering molecular signaling for bone formation and restoring sinonasal homeostasis through improved aerodynamics, this intervention may provide a comprehensive solution for chronic sinonasal inflammation beyond simple mechanical expansion.

## 1. Introduction

Maxillary deficiency in pediatric patients is frequently associated with constriction of the upper airway, contributing to chronic sinonasal inflammation and obstructive sleep-disordered breathing. Although conventional rapid maxillary expansion (RME) effectively increases transverse dental arch width, it does not fully address the complex three-dimensional skeletal dysmorphology or the associated functional and inflammatory alterations within the nasal airway.

The interrelationship between craniofacial morphology, airway patency, and respiratory function has long been a central focus in orthodontics and dentofacial orthopedics. Observational and imaging-based studies have demonstrated significant associations between airway adequacy, head posture, and craniofacial structure in growing individuals, indicating that reduced airway dimensions are linked to characteristic skeletal and postural adaptations [1,2,3,4]. Three-dimensional imaging further supports these findings, showing that diminished airway volume is often accompanied by altered mandibular positioning and craniofacial imbalance, underscoring the importance of airway considerations in orthodontic diagnosis and treatment planning [5,6].

At the molecular level, the therapeutic efficacy of the RAMPA system transcends simple physical displacement, anchoring itself in the principles of cellular mechanotransduction. The anterosuperior force vectors delivered by the appliance generate specific mechanical strain patterns across the circummaxillary sutures, which serve as primary biological interfaces. Previous studies have established that such tensile stress acts as a potent stimulus, inducing the upregulation of Bone Morphogenetic Protein-2 (BMP-2) and other osteogenic factors within the sutural microenvironment [7,8,9,10]. By identifying these highly stressed regions as the ‘BMP-2 TRIGGER ZONE,’ our framework bridges the gap between macroscopic orthopedic loading and microscopic bone remodeling. This mechanotherapeutic signaling not only facilitates rapid skeletal adaptation but also initiates a cascade of physiological improvements. Specifically, by reconfiguring the structural boundaries of the nasal cavity, these molecular triggers lay the foundation for enhanced aerodynamic performance and the subsequent resolution of chronic inflammatory markers, such as TNF-α, through restored sinonasal homeostasis.

Among the contributors to airway compromise, sinonasal pathology plays a critical and clinically relevant role. Radiological opacification of the paranasal sinuses typically reflects inflammatory processes—such as acute or chronic rhinosinusitis, mucosal edema, or mucus retention—rather than fixed skeletal obstruction [11,12,13]. In pediatric populations, chronic rhinosinusitis is characterized by persistent mucosal inflammation, impaired mucociliary clearance, obstruction of sinus ostia, and reduced aeration [14,15]. These inflammatory changes reduce effective air-filled volume and increase airway resistance, thereby contributing to functional nasal obstruction [12,15,16]. Importantly, because these processes are potentially reversible, they introduce a dynamic component to airway limitation that may interact with orthopedic interventions.

Maxillary expansion has long been employed to correct transverse deficiencies associated with maxillary hypoplasia. Early investigations by Derichsweiler [17] and Haas [18] demonstrated that RME improves not only maxillary width but also nasal respiration. Subsequent developments introduced slow maxillary expansion (SME) to reduce adverse effects associated with high-force protocols. In 1977, Mew proposed semi-rapid maxillary expansion (sRME), a hybrid approach balancing biomechanical efficiency with physiological adaptation [19]. This protocol has since demonstrated stable skeletal and dental outcomes across a broad pediatric age range [20].

Beyond dentofacial correction, maxillary expansion influences the nasal airway and paranasal sinus system. Structural modifications of the nasal cavity alter airflow patterns, airway resistance, and ventilation dynamics, which are closely linked to craniofacial growth and respiratory physiology [21,22,23]. Imaging studies have shown increases in nasal cavity width following expansion, particularly at the level of the inferior turbinates, although regional variations exist due to anatomical constraints near the ethmoid and sphenoid sinuses e.g., ref. [24].

Advances in cone-beam computed tomography (CBCT) and low-dose computed tomography have enabled precise volumetric assessment of the sinonasal complex, facilitating objective evaluation of treatment-induced changes. Using such techniques, RME has been extensively studied, with evidence demonstrating significant increases in upper airway volume and reductions in nasal airway resistance [16,21,25]. Husson et al. [26] further reported three-dimensional airway changes following maxillary protraction, establishing a precedent for protraction-induced airway remodeling. Notably, improvements in airflow have been observed even in cases with modest geometric changes, suggesting that functional benefits extend beyond simple dimensional enlargement.

Despite these advances, conventional expansion and protraction approaches remain limited in their ability to precisely control the direction of skeletal displacement and its downstream functional effects. Consequently, recent attention has shifted toward understanding how orthopedic interventions influence not only structural outcomes but also the functional and microenvironmental conditions within the nasal cavity.

**Figure 1:**
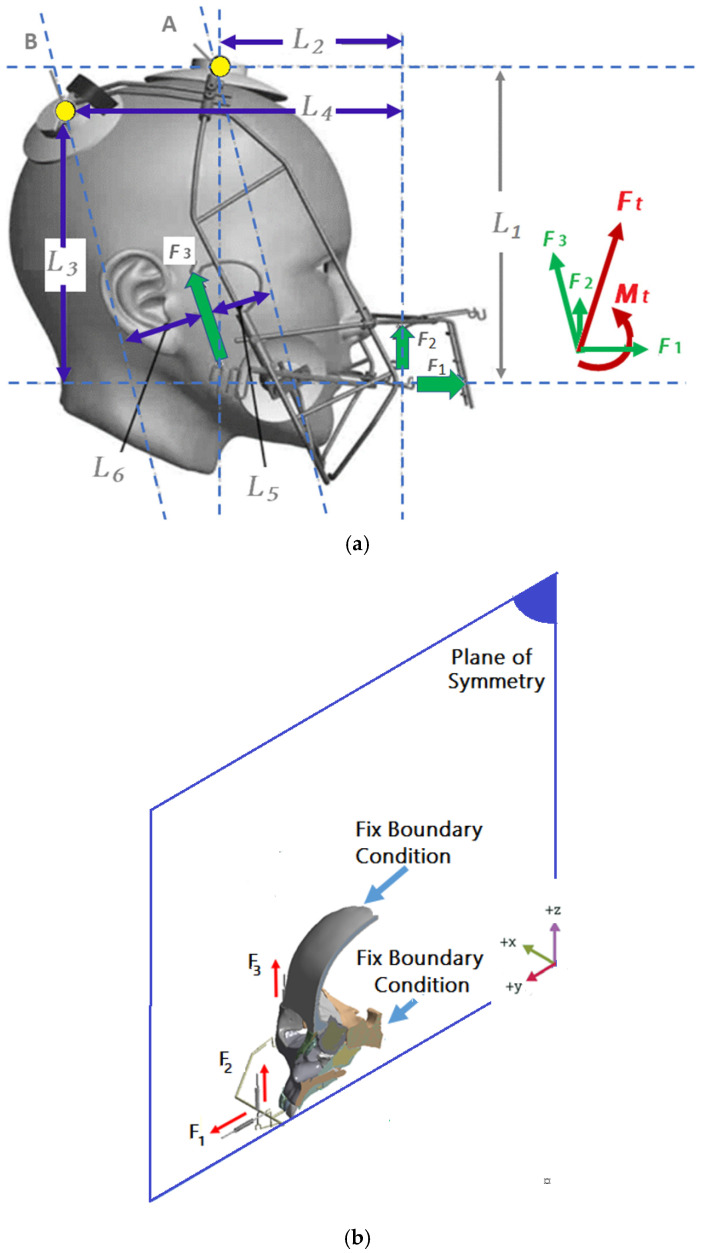
(**a**) Schematic demonstration of RAMPA including external forces; (**b**) External forces and boundary conditions applied to the FEM simulations.

In this context, the Right Angle Maxillary Protraction Appliance (RAMPA) represents a biomechanical advancement in maxillary orthopedic therapy (Figure 1). By combining semi-rapid maxillary expansion with controlled anterosuperior force vectors, RAMPA enables simultaneous transverse expansion and forward displacement of the maxilla [27,28,29,30,31]. Finite element analyses have demonstrated that this force system promotes coordinated forward and upward translation of the maxillocranial complex, optimizes stress distribution across circummaxillary sutures, and minimizes undesirable dental effects [27,28,29,30,31]. Furthermore, recent applications of finite element modeling continuously highlight its critical utility in evaluating complex biomechanical force systems and optimizing treatment mechanics in contemporary orthodontics [32]. Clinical and volumetric studies further indicate that such displacement increases nasal cavity dimensions and reduces airway resistance [33,34,35,36], with mathematical models and computational fluid dynamics (CFD) analyses suggesting enhanced airflow distribution and sinonasal ventilation [36,37,38]. While the computational fluid dynamics (CFD) approach offers significant potential for non-invasively assessing nasal aerodynamics and therapeutic outcomes, recent critical evaluations have increasingly emphasized the essential need to address methodological discrepancies and inherent limitations to enhance its clinical translational value [39].

Our previous work [40] demonstrated that RAMPA-induced remodeling reduces airflow resistance and increases airflow velocity within the nasal cavity, resulting in enhanced volumetric airflow under comparable respiratory effort. The significance of these macroscopic changes lies in their potential to modulate the nasal microenvironment.

The paranasal sinuses—including the maxillary, ethmoid, and sphenoid sinuses—drain mucus through narrow ostia, making effective clearance highly sensitive to local airflow conditions. Mucus exhibits non-Newtonian shear-thinning behavior, characterized by high viscosity at low shear rates and reduced viscosity under increased shear stress. This rheological property is fundamental to mucociliary clearance and airway health [35,36,41,42,43]. Increased airflow velocity is therefore expected to elevate shear forces, facilitating mucus transport and reducing stagnation.

In addition to steady airflow effects, respiration generates oscillatory flow patterns that promote bidirectional ventilation through sinus ostia. These dynamic flow conditions enhance air exchange and prevent stagnation while producing cyclic shear stress variations that may synergistically improve mucus mobilization [37,38]. Furthermore, airflow-induced mechanical stimuli may support mucosal hydration, epithelial function, and secondary clearance mechanisms alongside ciliary activity [44,45,46].

Collectively, these findings support a broader mechanistic framework in which orthodontic interventions act as mechanotherapeutic modulators of the nasal microenvironment. This framework integrates airflow dynamics, mucus rheology, clearance efficiency, and inflammatory processes. Impaired mucus clearance is a key driver of chronic sinonasal inflammation, as mucus stasis promotes microbial proliferation and sustained immune activation. Therefore, interventions that enhance airflow and mucus transport may contribute to resolving chronic inflammatory conditions.

Despite this emerging perspective, limited research has examined how baseline sinonasal inflammatory status—such as sinus opacification—interacts with orthopedic treatment. Because such conditions represent reversible soft-tissue obstruction rather than fixed anatomical constraints, their response to controlled maxillary protraction warrants systematic investigation.

Accordingly, the present study evaluates RAMPA therapy as a mechanotherapeutic intervention capable of inducing structural remodeling that translates into functional and pathophysiological changes within the sinonasal system. Using CBCT-based volumetric analysis, this retrospective cohort study assesses changes in the combined nasal cavity and paranasal sinuses following RAMPA therapy. Additionally, observed changes are compared with established growth baselines [47] to clarify the role of mechanotherapeutic maxillary remodeling in restructuring the sinonasal complex and modulating its functional and inflammatory state in pediatric patients.

## 2. Materials and Methods

### 2.1. Ethical Statement and Sample Size Rationale

This study utilizes anonymized retrospective pediatric patient data to construct patient-specific computational FEA and CFD models. The original CT data had been obtained for diagnostic purposes in accordance with the standard of care prior to this study. Due to the purely in silico nature of this analysis and the use of de-identified, non-identifiable retrospective data, direct IRB approval for this specific modeling study was determined not to be required according to institutional guidelines. Consequently, informed consent was exempted. The study was conducted in adherence to the principles of the Declaration of Helsinki.

A representative pediatric patient case, reflecting typical clinical and skeletal characteristics, was utilized to establish the reference geometry. As this is a mechanics-based deterministic computational study focused on elucidating fundamental biomechanical and aerodynamic mechanisms, a statistical power analysis for sample size justification was not applicable. Instead, model fidelity was ensured through high-resolution mesh validation and verification of established FEA/CFD solvers.

### 2.2. Data Integration Strategy

This integrative framework utilizes two distinct data sources to characterize the RAMPA mechanism and its clinical impact. The deterministic computational modeling (FEA/CFD) is derived from a single, high-resolution CT scan (602 × 328 pixels) to establish a high-fidelity representative model for mechanistic elucidation. Separately, the statistical validation of volumetric outcomes (Section 3.3) is performed using a distinct clinical cohort of 20 pediatric patients who underwent RAMPA treatment, with pre-and post-treatment analyses based on standardized CBCT scans. This disconnect in data origin between the in silico models and the population-level analysis is a known limitation that is addressed in Section 4.

### 2.3. Study Design Overview: Integrated Causal-Cascade Master Diagram

**Figure 2:**
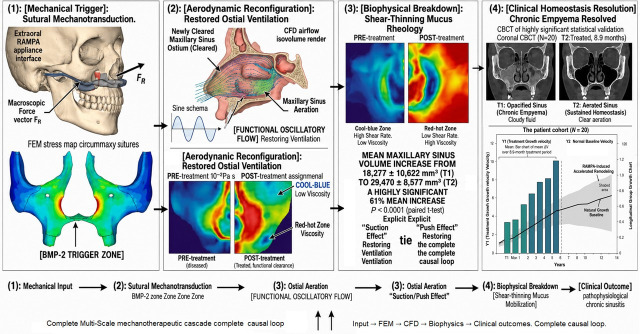
Integrated multi-scale mechanotherapeutic causal-cascade blueprint for pediatric chronic sinusitis resolution.

As shown in Figure 2, the present study is presented in the form of an Integrated Causal-Cascade Master Diagram to elucidate the complete mechanistic pathway through which RAMPA therapy restores sinonasal homeostasis in pediatric patients with maxillary deficiency and concomitant chronic sinusitis (empyema). This diagram provides a holistic multi-scale blueprint, visually connecting raw physical force triggers to biophysical modulation and ultimately to pathophysiological resolution.

### 2.4. Finite Element Modeling and Mechanotherapeutic Stress Mapping

In order to ensure highly reliable and high-fidelity mechanotherapeutic simulations, a three-dimensional (3D) finite element (FE) model of a pediatric human skull was utilized, following the validated geometric framework and boundary conditions established in previous research [31]. The CAD model, originally constructed from a 3B Scientific skull replica (Model: 9982-1000069) and supplemented by high-resolution anatomical data, incorporates detailed representations of cortical and cancellous bone structures to maximize predictive accuracy. To ensure physiological relevance, periodontal ligaments (PDLs) with thicknesses within the documented ranges [48,49,50,51] and craniofacial sutures with thicknesses between 0.1 and 0.5 mm [52,53] were meticulously modeled. Although these sutures are not visually discernible at a macroscopic scale due to their submillimeter thickness, they are fully incorporated into the FE model to capture their precise mechanical behavior.

**Figure 3:**
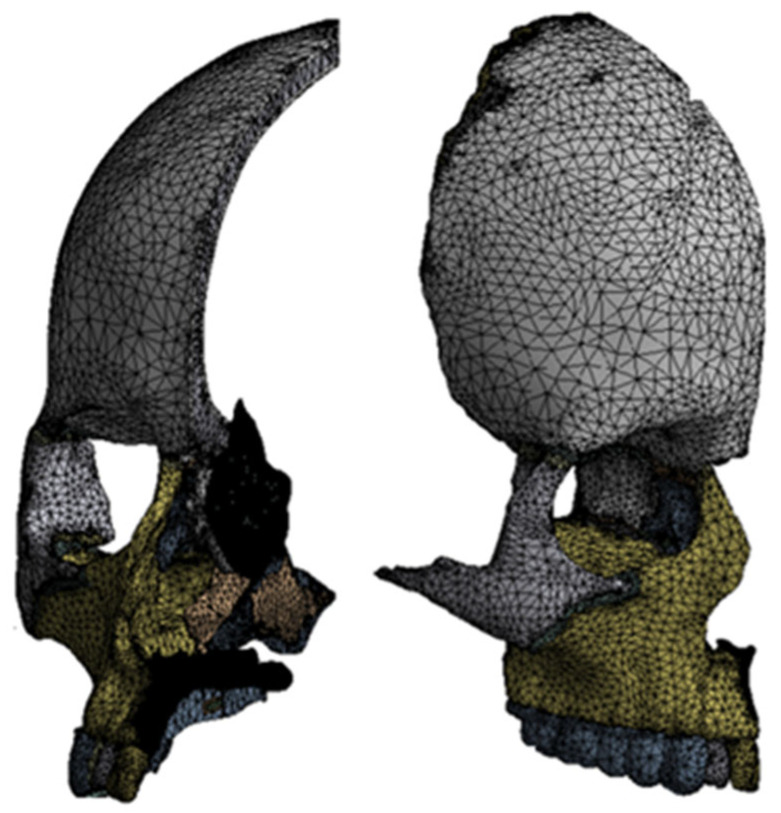
Mesh arrangement used in FEM simulation from two different views.

Finite element analysis was performed using ANSYS Workbench 17.2 (Ansys Inc., Canonsburg, Penn, USA). The final computational mesh (Figure 3) comprised 739,000 tetrahedral elements and 1,300,000 nodes, a configuration that has reached an acceptable level of mesh independence [31]. Element sizes were specifically tailored according to the anatomical region; finer meshes were assigned to sutural areas—with at least five elements distributed across their thickness—to accurately capture local stress and strain gradients under loading. The Material properties applied to the FEM simulations are listed in Table 1.

The Right Angle Maxillary Protraction Appliance (RAMPA) and the RAMPA Oral Appliance (ROA) were digitally interfaced with the maxillary structure to simulate clinical loading conditions (Figure 1). Mirroring the clinical protocol, a total of six force vectors were applied through elastic bands: two horizontal (anteriorly directed) and four vertical (superiorly directed) forces. Based on previous tensile testing [28], the magnitudes were set at F_1_ = 2.94 N, F_2_ = 1.44 N, and F_3_ = 4.0 N (Table 2). Boundary conditions were established by constraining the cranial base and the occipital region to prevent rigid body translation while allowing for physiological sutural deformation.

The resulting von Mises stress distribution, induced by the prescribed forces (F1 = 2.94 N, F2 = 1.44 N, and F3 = 4.0 N), was mapped across the circummaxillary sutures—specifically targeting the pterygomaxillary, zygomaticomaxillary, and midpalatal sutures—to define the BMP-2 TRIGGER ZONE. A definitive von Mises stress threshold of 3.5 MPa was established based on mechanics-based literature investigating craniofacial suture mechanobiology, particularly modeling work that derived quantitative thresholds from experimental data [54]. Mechanical stresses exceeding this defined numerical threshold are assumed sufficient to trigger cellular mechanotransduction, consistent with mechanistic studies showing how definitive mechanical stimuli induce cellular activity and upregulate specific osteogenic factors, such as BMP-2 and BMP-4, within fibrous tissues [7,8]. Consequently, the mapped trigger zones provide a rigorous, comprehensive evaluation of how the macroscopic mechanical triggers align precisely with recognized cell-level responses needed for effective craniofacial remodeling [10].

**Table 1:** Material properties applied to the FEM simulations.

	Young’s Modulus (Mpa)	Poisson’s Ratio
Cortical bone	13,800 Mpa	0.26
Cancellous bone	1370 Mpa	0.3
Resin	3543 Mpa	0.3
Stainless steel	193,000 Mpa	0.31
Teeth	18,600 Mpa	0.31
Periodontal ligament	50 Mpa	0.49
Midpalatal suture (MPS)	50 Mpa	0.49
Sutures	30 Mpa (Case 1)	-
	50 Mpa (Case 2)	0.49
	80 Mpa (Case 3)	0.49
	13,800 Mpa (No suture; Case 4)	0.49

**Table 2:** Forces and dimensions of RAMPA used for the present case study.

Forces (N)			Dimensions (mm)					
$\left|\vec{F_{1}}\right|$	$\left|\vec{F_{2}}\right|$	$\left|\vec{F_{3}}\right|$	$L_{1}$	$L_{2}$	$L_{3}$	$L_{4}$	$L_{5}$	$L_{6}$
2.94	1.44	4.0	190	105	160	185	35	50

The resultant force (F_t_) and moment (M_t_) acting on the device (Figure 1) can be calculated as follows:

(1) $$\vec{F_{t}}=\vec{F_{1}}+\vec{F_{2}}+\vec{F_{3}}$$

(2) $$\vec{M_{t}}=\vec{M_{A}}+\vec{M_{B}}$$

where

(3) $$\left|M_{A}\right|=F_{1}L_{1}+F_{2}L_{2}+F_{3}L_{5}\;\left|M_{B}\right|=F_{1}L_{3}+F_{2}L_{4}+F_{3}L_{6}$$

### 2.5. Air Circulation Simulation in Nasal Passages Using CFD

**Figure 4:**
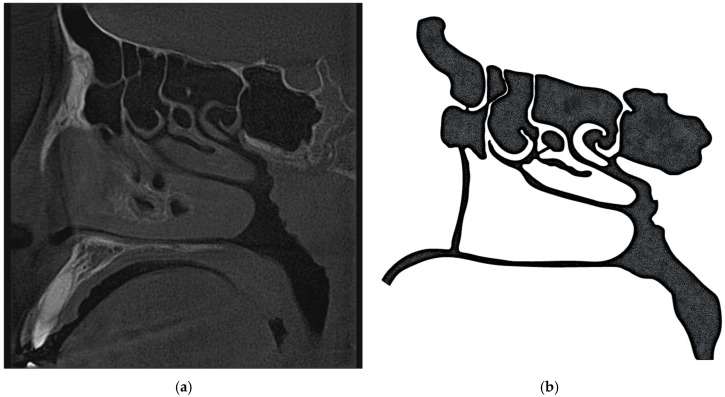
(**a**) CT-scan images of a patient; (**b**) CFD domain generated based on the CT-scan image.

A CT-Scan image (Figure 4a) of nasal passages has been used to generate the geometry of a base model for 3D CFD of the airway and paranasal sinuses (Figure 4b) [33]. The model included the nasal cavity, nasopharynx, and the maxillary, frontal, ethmoid (anterior and posterior), and sphenoid sinuses. The CFD domain extends from the nostrils to a region near the throat (pharynx). In addition to the baseline geometry, a secondary model has also been created by scaling the base geometry by a factor of 1.02 (expansion of 2%). This secondary model has been used for the comparison between mucus discharge between the base geometry and the expanded one.

The mesh included meshed 790,000 polyhedral cells, with a maximum cell size of 0.7 mm and prism layers with a resolution of 0.06 mm and an expansion ratio of 1.2. To mimic real case scenario, the boundary conditions applied to the CFD model were a uniform flow velocity at the throat (for inhale and exhale push) and a free inlet with atmospheric pressure at the nostrils. In all simulations, unsteady RANS simulations with a k–ε turbulence model have been used. The simulations were performed using a fixed time step of 0.075 s, with 10 inner iterations per step applied.

#### 2.5.1. CFD Simulations Using Newtonian Fluid Assumption

The CFD modeling consists of Volume of Fluid (VOF) simulations. A set of simulations have been performed with Newtonian assumption and the other set with non-Newtonian assumption for mucus. Air and mucus densities have been set to fixed values of 1.184 and 1000 $kg/m^{3}$. Dynamics viscosity for water and mucus are fixed at $1.85\times 10^{-5}$ Pa·s and 12 Pa·s.

#### 2.5.2. Biophysical Viscosity Breakdown: Shear-Dependent Mucus Rheology

For the non-Newtonian simulations, mucus is a shear-thinning, viscoelastic biological fluid, for which a generalized non-Newtonian representation is commonly adopted in CFD simulations. In the present framework, the apparent viscosity is expressed as a function of shear rate, and the Carreau–Yasuda model (Equation (4)) is used [34,35] due to its ability to capture both low- and high-shear viscosity plateaus.

(4) $$\mu \left(\dot{\gamma }\right)=\mu_{\infty }+\left(\mu_{0}-\mu_{\infty }\right){\left[1+{\left(\lambda \dot{\gamma }\right)}^{2}\right]}^{\frac{n-1}{2}}$$

For human respiratory mucus, physiologically realistic parameter ranges reported in the literature include a zero-shear viscosity $\mu_{0}$ on the order of 10–100 Pa·s, an infinite-shear viscosity $\mu_{\infty }$ approaching that of water ($10^{-3}$ to $10^{-2}\;$ Pa·s), a power-law index of 0.15 < n < 0.35 indicating strong shear-thinning behavior, and a characteristic time constant λ typically spanning 1–100 s. A representative parameter set frequently employed in airway simulations is $\mu_{0}=$ 12 Pa·s and ${\;\mu }_{\infty }=$ 0.0007 Pa·s (water at 37 °C), λ = 40 s, and n = 0.25, which reproduces experimentally observed viscosity reductions over several orders of magnitude across physiological shear rates [34,35]. Within the Carreau formulation, the parameter λ is commonly referred to as the relaxation time, although in this context it serves as a phenomenological descriptor of the transition between Newtonian and shear-thinning regimes rather than a full viscoelastic stress relaxation parameter. Physically, relaxation time represents the characteristic timescale over which the internal microstructure of the fluid—primarily the entangled mucin polymer network in mucus—recovers after deformation. A larger λ implies prolonged structural memory and earlier onset of shear-thinning at lower shear rates, while a smaller λ corresponds to rapid microstructural rearrangement and more Newtonian-like behavior (*λ* = 40 s means that the mucus has strong internal structure i.e., mucin network remembers deformation and hence demonstrates viscoelastic behavior). This transition is governed by the dimensionless group λγ˙, such that for $\dot{\gamma }$ ≪ 1/λ, the viscosity approaches $\mu_{0}$ whereas for $\dot{\gamma }$ ≫ 1/λ, the viscosity asymptotically approaches $\mu_{\infty }$.

Although human mucus exhibits clear viscoelastic behavior, including elastic recoil and time-dependent stress relaxation, the Carreau model remains a pragmatic choice in CFD platforms, due to its numerical robustness and reduced computational cost. For sinus and airway simulations, CFD simulations with an adequate first-order approximation of flow resistance, wall shear distribution, and mucus transport under varying airflow conditions are considered accurate. Nevertheless, careful parameter selection and sensitivity analysis are essential, particularly with respect to $\mu_{0}\;$ and n, as these strongly influence predicted viscosity fields and flow behavior. The adopted values should be validated against experimental rheometry data where available, especially in pathological conditions (e.g., chronic rhinosinusitis or cystic fibrosis), where mucus rheology may deviate substantially from healthy baseline properties.

### 2.6. Statistical Validation of Clinical Homeostasis and Group Longitudinal Growth

#### 2.6.1. Patient Cohort and CBCT Acquisition

To validate the hypothesized mechanotherapeutic cascade, a retrospective analysis was performed on a pediatric patient cohort.

1. Inclusion Criteria: The study included 20 pediatric patients presenting with pre-treatment Opacified sinuses, indicative of empyema and mucostasis across circummaxillary sutures.
2. Longitudinal CBCT Protocol: Longitudinal Cone-Beam Computed Tomography (CBCT) scans were utilized: Pre-treatment (T1) and a post-treatment (T2) scan obtained after a definitive treatment period. Standardized scanning parameters were specifically optimized for the Alphard-3030 system (Asahi Roentgen Ind. Co., Ltd., Kyoto, Japan) to reduce radiation dose to the pediatric population while maintaining high diagnostic image quality. The acquisition protocol utilized 80 kVp and 5 mA, resulting in an image resolution of 0.3 mm voxel size. The field of view (FOV) was standardized to encompass the entire craniofacial complex from the frontal sinus superiorly to the hyoid bone inferiorly.

#### 2.6.2. Image Analysis and Volumetric Quantification

To ensure objective quantification, the acquired CBCT DICOM files were analyzed using 3D imaging software (Mimics Innovation Suite; Materialise version 27.0, N.V., Leuven, Belgium). Quantitative volumetric changes of the nasal cavity and paranasal sinuses (mm^3^) were automatically calculated based on the segmented geometries. The air-filled spaces were segmented using a calibrated threshold range of −1000 to −625 Hounsfield Units (HU). This threshold setting was validated by both previous studies for pediatric airway analysis and our own pilot study, which included internal sampling of airspace pixel intensities to accurately delineate the interface between respiratory cavities and surrounding soft tissues. To eliminate memory bias, these cases were re-analyzed by the primary investigator and an additional calibrated observer after a two-week interval. Intraclass Correlation Coefficient (ICC) values for both intra- and inter-observer reliability were found to be above 0.90, confirming excellent reproducibility of the landmark identification and the manual refinement-inclusive segmentation protocol. Quantitative volumetric changes of the nasal cavity and paranasal sinuses (mm^3^) were automatically calculated based on these validated segmented geometries. Group-level transition from Opacified Sinus (T1) to Aerated Sinus (T2) was verified based on these quantified volumes.

#### 2.6.3. Statistical Analysis and Growth Velocity vs. Baseline

Statistical analyses were performed to validate the multi-scale causal cascade.

1. Volumetric Significance: A paired t-test was performed to determine the statistical significance of the mean volumetric increase from T1 to T2, with the significance threshold set at *p* < 0.0001.
2. Growth Velocity Comparison: A comparative longitudinal growth chart was constructed at the cohort level. The patient group’s mean Volumetric Change (∆V, representing growth velocity) over the mean treatment period was contrasted against established natural growth baselines reported in the literature [46]. A shaded area on the growth chart illustrates the difference.

## 3. Results

This study factually establishes an integrated multi-scale causal blueprint demonstrating craniofacial mechanics actively trigger a pathophysiological cascade leading to restoration of sinonasal homeostasis. The research sequentially validates findings for the Mechanical Trigger, Airflow Reconfiguration and Biophysical Nonlinear Viscosity, and concludes with final Clinical Homeostasis and Accelerated Growth outcomes.

### 3.1. Mechanical Trigger and Sutural Mechanotransduction

**Figure 5:**
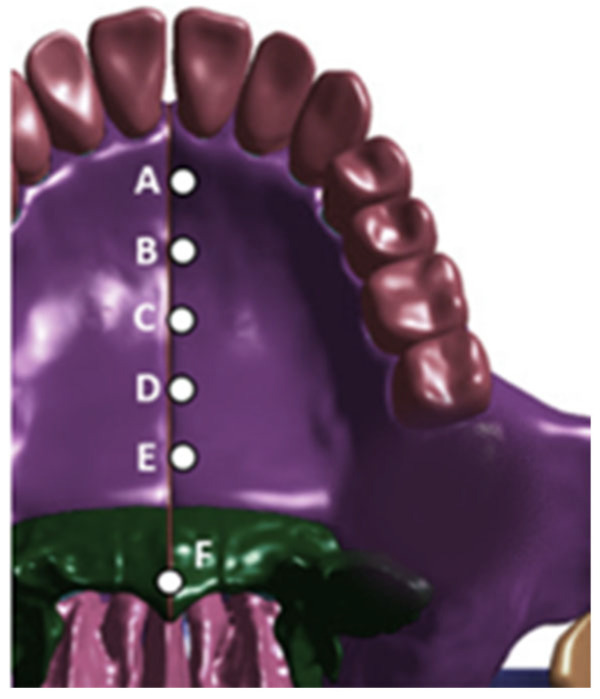
Points A–F on the MPS for displacement assessment of the craniofacial system: A: near the incisive foramen; E: point near the palatine bone; points B–D placed equidistantly on A–E line.

**Figure 6:**
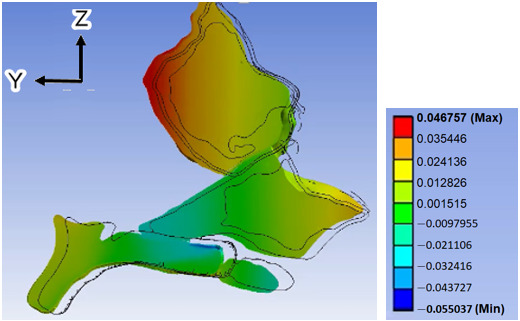
Midpalatal suture Z displacements (mm) for RAMPA combined with Hybrid [55]. Reproduced with permission from [55]; published by Taylor & Francis, 2025.

**Figure 7:**
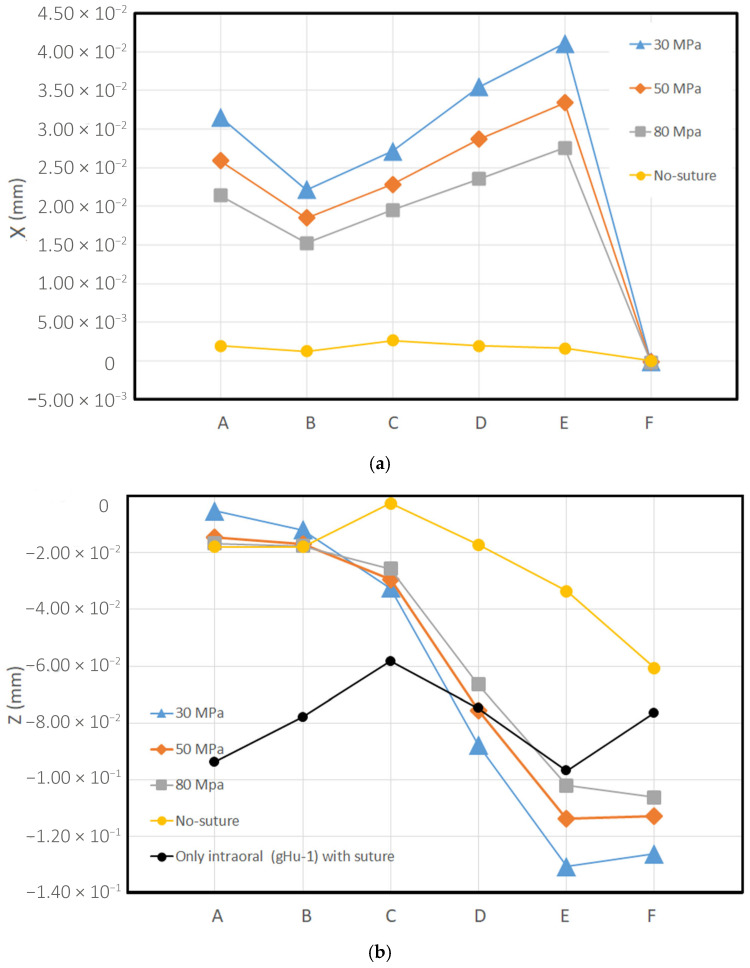
Midpalatal suture displacements for RAMPA combined with gHu-1 for different suture material properties; (**a**) in X direction, (**b**) in Z direction [31]. Reproduced from [31] under the Creative Commons Attribution (CC BY) license.

FEM analysis reveals that the extraoral appliance transmits anterosuperior force vectors creating anterosuperior maxilla deformation resulting in anterosuperior protraction across different ROAs including Vompress [30], gHu-1 [33], and Hybrid [55]. Displacement study factually indicates significant impact for points A–F on the Midpalatal suture (MPS) (Table 3 and Table 4, Figure 5 and Figure 6). This protraction behavior factually demonstrates a consistent general result robust across individuals with different suture stiffness levels [31] (Figure 7a,b).

### 3.2. CFD Simulation of Air Circulation Inside Nasal Passages

#### 3.2.1. Impact of Geometrical Expansion on Flow Field Inside the Nasal Cavity (Single Phase)

**Figure 8:**
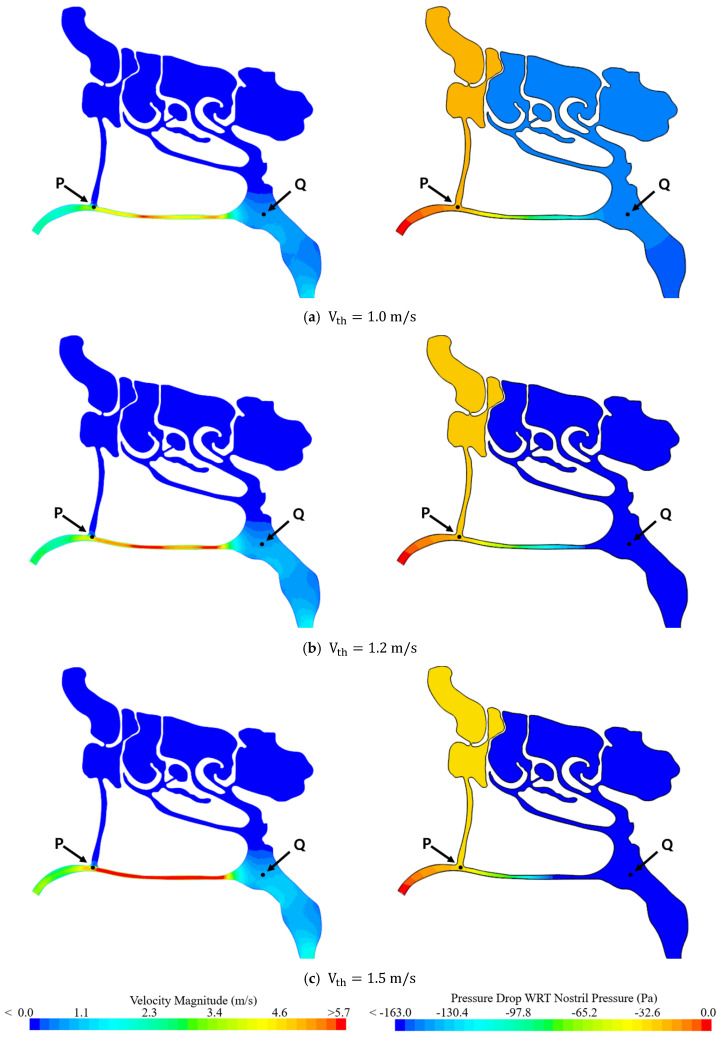
Velocity and pressure distribution of the nasal passage for different throat air velocities.

Summarizing previous research, this subsection investigates how cavity expansion increases airflow velocity and local pressure within the nasal cavity. CFD simulations utilized distinct throat boundary velocities $V_{\mathrm{th}}$ of {1.0, 1.2, 1.5} m/s, reflecting inherent variations across expanded anatomies, while air was introduced freely through the nostrils (satisfying mass conservation). For $V_{\mathrm{th}}$ = 1.0 m/s, this equated to a volumetric flow rate of approximately 4 L/min, consistent with the literature [56]. These simulations evaluated domain-wide pressure and velocity distributions, focusing on sample points P and Q (Figure 8).

Significant spatial variations in pressure loss are observed along the nostril-to-larynx flow path, particularly distinguishing the anterior frontal sinus channels from the posterior pathways connected to the ethmoidal and sphenoidal sinuses. This results in a pronounced pressure differential between points P and Q. Moreover, flow velocity remains near zero within channels linked to sinuses with stagnated flow. Rheologically, this confirms that sinus mucus (pus) discharge is governed by both ciliary action and mucus viscosity, thereby underscoring the critical importance of accurate viscosity parameters for CFD settings, which are detailed in the subsequent section.

#### 3.2.2. Impact of Rheology Modeling on Mucus Discharge in Two-Phase Flow Simulation

**Figure 9:**
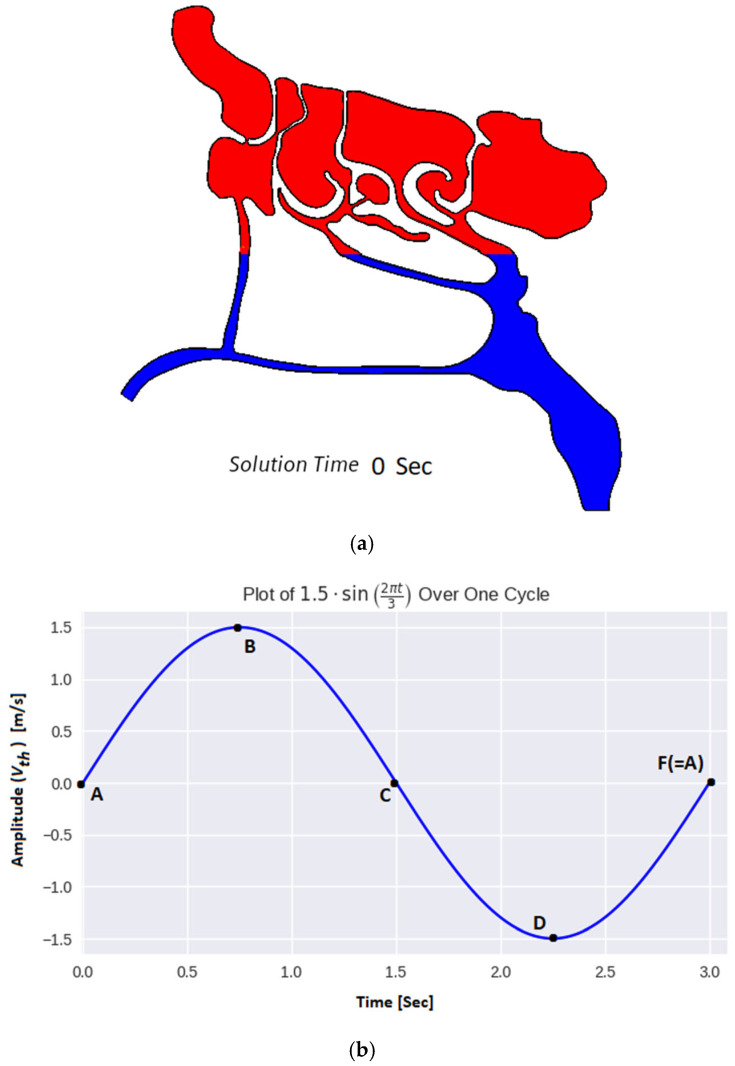
(**a**) Initial filling of mucus in the CFD domain; (**b**) Velocity profile of one inhale-exhale cycle at throat location (Points A–F are phase markers of the breathing cycle.

Within this subsection, three distinct CFD simulations have been performed: two with Newtonian flow with constant dynamic viscosity ($\mu_{pus}=$ 12 Pa·s and $\mu_{water}=$ 0.0007 Pa·s) and one with non-Newtonian viscosity using the Carreau–Yasuda model as detailed in Section 2.5.2. The CFD domain was assumed to be initially partially filled with mucus, as shown in Figure 9a. All simulations employed a sinusoidal velocity profile corresponding to 20 breaths per minute [56] prescribed at the throat as follows:

(5) $$V\left(t\right)=V_{th}\sin\left(2\pi ft\right)$$

This throat velocity oscillates sinusoidally over 3 s, representing one complete inhale-exhale cycle, as depicted in Figure 9b.

**Figure 10:**
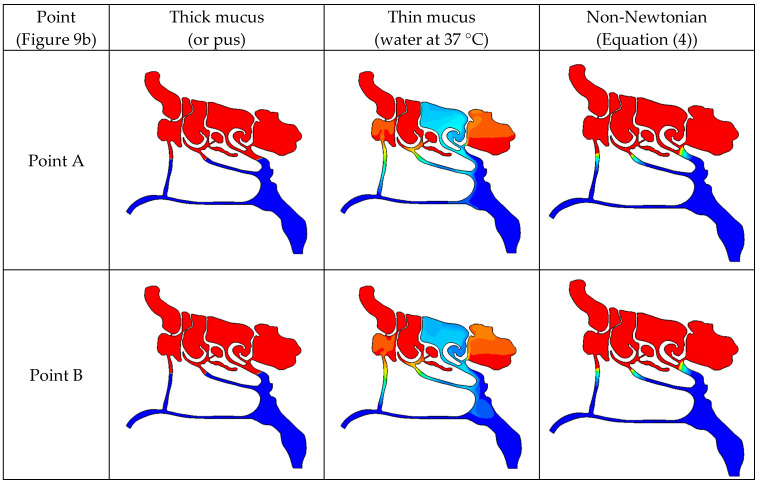

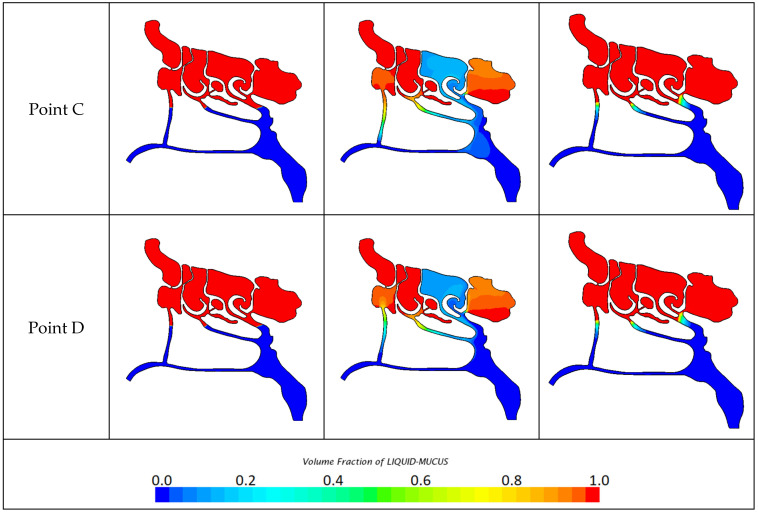
Impact of viscosity model on mucus discharge during one inhale-exhale cycle (t = 6 s to 9 s).

**Figure 11:**
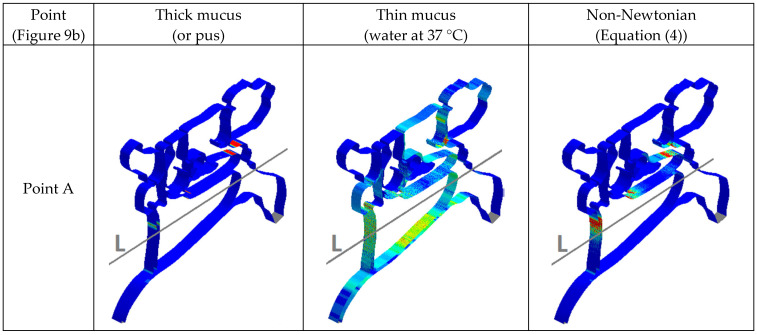

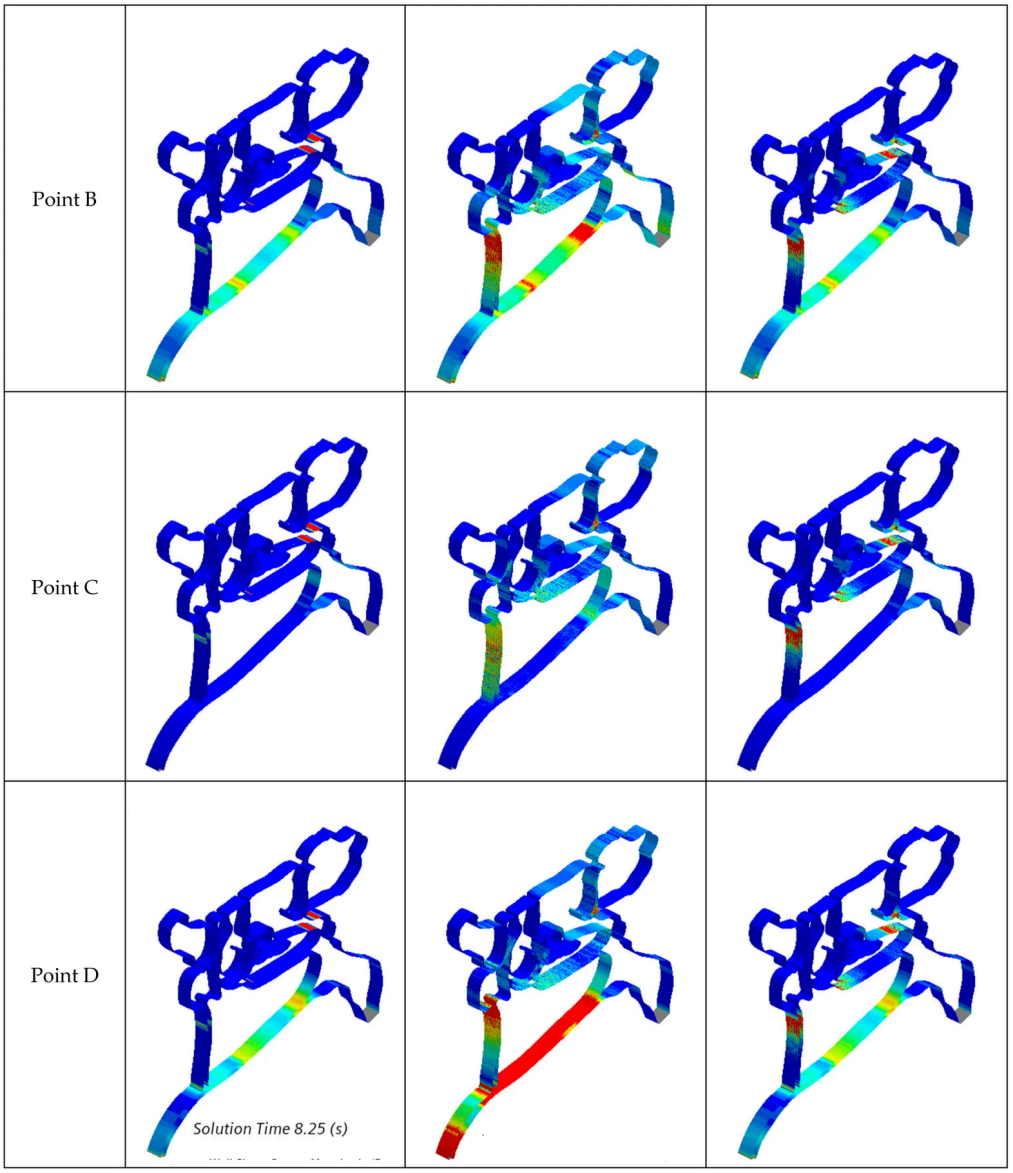

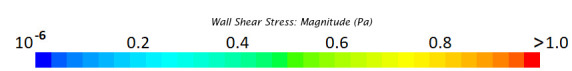
Impact of viscosity model on wall shear stress during one inhale-exhale cycle (t = 6 s to 9 s).

Table 5 and the simulation results (Figure 10 and Figure 11) confirm that realistic mucus viscosity assumptions are essential for accurate mucus discharge prediction. Specifically, the non-Newtonian Carreau–Yasuda model (Equation (4)), which accounts for mucus’s shear-thinning properties, captures inherently non-linear behavior much more realistically and accurately than assuming constant, water-like viscosity near the wall [57]. The differences in discharge characteristics stemming from these viscosity models are clearly visible in the simulated mucus volume fraction variations observed over one complete breathing cycle between t = 6 s and 9 s. The constant high-viscosity Newtonian model, lacking shear-thinning effects, resulted in mucus sticking to the nasal cavity walls and exhibiting no movement at all. Conversely, the constant low-viscosity, water-like model yielded unrealistically rapid mucus discharge rates. In contrast, applying the non-Newtonian shear-thinning assumption allowed mucus discharge to proceed in a gradual and realistic manner.

The interpretation of wall shear stress (WSS) in air-mucus simulations depends on local phase composition, as its physical significance differs among air-filled, mucus-filled, and mixed interface regions. In air-dominated zones, high WSS reflects strong velocity gradients and efficient ventilation, while low WSS indicates weak flow or recirculation. Conversely, in mucus-dominated regions governed by shear-thinning rheology, high WSS corresponds to local deformation and viscosity reduction, whereas low WSS results in stagnant, highly viscous mucus prone to retention.

The mixed interface regions are functionally crucial, as WSS here represents the direct transfer of aerodynamic shear to the mucus layer. High WSS promotes mucus mobilization, acting as a key mechanism for clearance between sinus cavities and the main airway. Low WSS signifies insufficient shear transfer, creating bottlenecks that limit transport despite partial ventilation. Accurately interpreting the WSS field thus requires recognizing these phase-dependent meanings to properly assess ventilation efficiency, retention zones, and regions where shear-thinning facilitates clearance.

Establishing the necessity of non-Newtonian modeling for mucus clearance, we compared mucus transport between base and expanded geometries. The expanded geometry simulates RAMPA therapy via a 5% nasal passage enlargement (1.05 expansion ratio). Due to the directional expansion, throat airflow velocity was increased by $1.05^{2}$ (equivalent to 10% increase). This model used an initial mucus volume of 3920 $mm^{3}$, keeping the relative initial mucus level constant. Identical non-Newtonian and k-ε turbulence models were used. Table 6 compares the impact of this expansion on mucus discharge.

Geometric comparison reveals that even a modest enlargement can meaningfully influence mucus transport dynamics. Although the expanded configuration (×1.05) increased initial mucus volume by approximately 6%, it produced a disproportionately larger improvement in discharge behavior. The discharged volume rose from 48 mm^3^ to approximately 61.8 mm^3^, corresponding to an increase in discharge rate from 1.42% to 1.57% with respect to total volume. This 13.8 mm^3^ increase represents a 29% improvement relative to the base geometry’s discharged volume. These findings indicate that small geometric modifications can reduce flow resistance and promote more effective mucus expulsion under the same viscosity model, highlighting the high sensitivity of clearance performance to structural variations.

### 3.3. Clinical Outcome: Empyema Clearing and Group Growth

**Figure 12:**
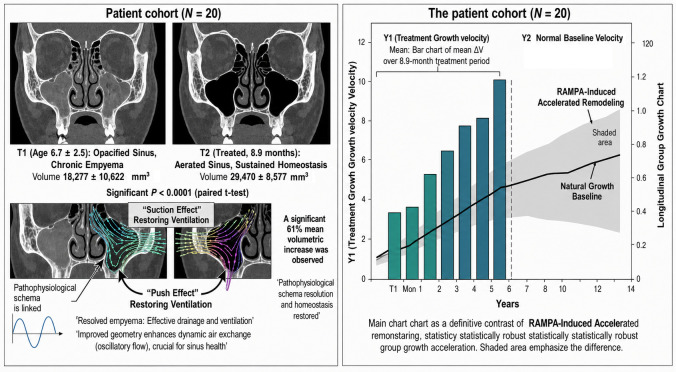
Clinical homeostasis resolution and longitudinal group growth acceleration.

Evaluation of the integrated causal blueprint suggests that biophysical homeostasis resolution may translate into pathophysiological improvement (Figure 12).

1. Cohort Baseline and Homeostasis Resolution: Clinical analysis of the patient cohort (n = 20; mean age 6.7 ± 2.5 years at T1) demonstrated favorable outcomes. An observable difference was noted between pre-treatment (T1) opacified sinuses, indicative of empyema and mucostasis, and post-treatment (T2) scans showing improved sinus aeration.
2. Volumetric Statistics and Shear-Dependent Viscosity Changes: Following a mean treatment period of 8.9 months, a significant 61% mean volumetric increase was observed (from 18,277 ± 10,622 mm^3^ at T1 to 29,470 ± 8577 mm^3^ at T2; *p* < 0.0001 paired t-test). The estimated difference between PRE (mucostasis risk, 10^3^ Pa·s) and POST (functional clearance, 10^−2^ Pa·s) conditions provides preliminary support for the shear-dependent rheology breakdown hypothesis derived from CFD-determined shear rates.
3. Group Growth Velocity Trends: Comparative longitudinal growth chart analysis indicates that the mean group growth velocity over the treatment period exceeded the established natural growth baseline [47]. This observation suggests a potential acceleration of growth velocity associated with the treatment.

## 4. Discussion

### 4.1. Mechanical, Aerodynamic, and Rheological Foundation for Homeostasis Restoration

Our integrated modeling confirms the mechanical efficiency of the RAMPA appliance. It successfully generates direct, directional force vectors and consistent maxilla protraction across varied interoral devices, even across varied sutural stiffness levels. This robust mechanical trigger is the primary catalyst for subsequent sinonasal remodeling.

Crucially, the CFD simulations reveal the aerodynamic mechanism behind clearance success. The complex physical meaning of wall shear stress (WSS) distribution cannot rely on magnitude alone; it must be interpreted using our phase-dependent methodology (air, mucus, and interface). In purely air-dominated zones, WSS primarily reflects ventilation efficiency. Conversely, in mucus-dominated upper cavities, WSS indicates local deformation and potentially mobilization due to shear-thinning; however, low WSS in these regions highlights bottlenecks where high-viscosity mucus remains stagnant and prone to retention.

The mixed (air–mucus interface) regions emerged as the functional gatekeepers controlling mucus exchange. High WSS in these interface zones signifies efficient momentum transfer from air to the mucus layer, directly driving shear-thinning and enabling detachment and potential mobilization. Overall, the accurate identification of functional clearance mechanisms requires this phase-dependent interpretation rather than simple volumetric velocity measurement.

The geometry comparison further demonstrates that even minimal structural modifications—a modest (5%) expansion—can produce a disproportionate enhancement in discharge performance and transport efficiency under identical rheological conditions. Small structural enlargements meaningfully reduce flow resistance and facilitate effective mucus mobilization, linking orthopedic displacement directly to biological function restoration.

### 4.2. Pathophysiological Resolution, Group Growth, and Accelerated Remodeling

The clinical cohort data (n = 20) definitively validates this multi-scale causal model. Our statistical analysis demonstrates a significant 61% mean volumetric increase, confirming skeletal remodeling far beyond simple case reports. This resolution is driven by a two-phase improvement mechanism: immediate obstruction relief through rapid empyema drainage and mucosal edema reduction, followed by long-term sustained volumetric gains driven by RAMPA-induced orthopedic displacement.

This robust clinical outcome is catalyzed by sutural mechanotransduction, which upregulates molecular markers like Bone Morphogenetic Protein-2 (BMP-2). This signaling likely activates osteoblast differentiation within critical ‘trigger zones’, accelerating sutural remodeling and driving the observed Group Growth Acceleration. Contrast against established natural developmental baselines [47] confirms that RAMPA does not merely track natural growth but actively accelerates complex restructuring to restore homeostasis.

### 4.3. Limitations and Future Directions

Despite the powerful mechanistic explanation provided by the cohort evaluation, certain limitations remain. As a proof-of-concept study, the sample size is relatively small (n = 20). While the clinical results are statistically robust, the absence of an untreated control group means spontaneous resolution cannot be entirely excluded, although the magnitude of change (61%) makes this unlikely. Furthermore, the “BMP-2 trigger zone” proposed in this study is a computational inference lacking direct biological validation. Additionally, several methodological constraints in our computational simulations and imaging analysis should be noted. First, the finite element analysis (FEA) relied on a standard, non-patient-specific pediatric skull replica model. While this framework is biomechanically validated, it may not fully represent the individual anatomical variations and specific sutural properties of our clinical cohort. Second, the computational fluid dynamics (CFD) model was derived from a single representative patient’s nasal geometry rather than being patient-specific to the clinical cohort, which limits direct individual correlation between aerodynamic predictions and clinical volumetric outcomes. Lastly, while our volumetric quantifications demonstrated high inter-rater reliability (ICC > 0.9), they were performed without a universally standardized segmentation protocol, potentially introducing subtle variability in boundary definitions. Future research should focus on larger prospective studies with matched control groups, patient-specific computational models, and in vivo measurements of molecular markers, such as BMP-2 upregulation in the trigger zones or pro-inflammatory cytokine downregulation (e.g., TNF-α), to further refine the cellular-level details of this multi-scale causal blueprint.

## 5. Conclusions

The present study successfully establishes an integrated multi-scale mechanotherapeutic cascade as a proof-of-concept, suggesting a potential “Chain of Causality” through which RAMPA therapy restores sinonasal homeostasis. By unifying macroscopic orthopedic forces with microscopic biophysical modulations, we provide a holistic blueprint for the treatment of pediatric sinonasal compromise.

Our findings confirm that the RAMPA appliance acts as a reliable mechanical trigger, delivering anterosuperior force vectors that initiate sutural mechanotransduction and subsequent craniofacial remodeling. This structural expansion modifies the nasal microenvironment by significantly increasing airflow velocity and restoring bidirectional oscillatory flow patterns. These aerodynamic reconfigurations provide the critical shear rates necessary to induce “Biophysical Viscosity Breakdown” through the shear-thinning behavior of respiratory mucus. This process transforms stagnant, high-viscosity mucostasis into mobilizable low-viscosity mucus, facilitating functional clearance and resolving chronic inflammatory states.

Quantitative evaluation across a patient cohort (n = 20) provides preliminary evidence supporting the clinical efficacy of this model, showing a significant 61% mean volumetric increase (18,277 ± 10,622 mm^3^ to 29,470 ± 8577 mm^3^; *p* < 0.0001) within an average treatment period of just 8.9 months. Furthermore, the attainment of “Group Growth Acceleration” compared to established natural growth baselines [47] confirms that mechanotherapeutic remodeling actively accelerates developmental velocities to restore physiological homeostasis.

In conclusion, this integrated framework—summarized in our Integrated Mechanotherapeutic Causal Cascade—offers a persuasive new paradigm for treating pediatric respiratory impairments by addressing both skeletal form and pathophysiological function simultaneously. These insights provide clinicians with a more effective, interdisciplinary pathway for managing craniofacial deficiencies and concomitant chronic sinusitis.

## Figures and Tables

**Table 3:** Midpalatal suture displacements for RAMPA combined with Vompress [30]. Reproduced with permission from [30]; published by Taylor & Francis, 2022.

Point	X (mm)	Y (mm)	Z (mm)
A	0.0428	0.2659	0.0395
B	0.0163	0.2341	0.0102
C	0.0052	0.2319	0.0153
D	0.0026	0.2301	0.0212
E	−0.0001	0.2304	0.0213
F	0.0001	0.2299	0.0108

**Table 4:** Midpalatal suture lateral displacements for RAMPA combined with g-Hu1 [33]. Reproduced from [33] under the Creative Commons Attribution (CC BY) license.

Point	X (mm)
A (near incisive foramen)	0.151
B	0.144
C	0.138
D	0.128
E (near palatine bone)	0.101

**Table 5:** Mucus (pus) discharge from the throat cross-section in the present CFD simulations under different viscosity assumptions.

Assumption	Viscosity (Pa·s)	Initial Volume of Mucus or Pus (mm^3^)	Volume of Discharged Mucus or Pus After 15 s
Thick mucus or pus	$\mu_{pus}=$ 12	3384	0
Thin mucus (water at 37 °C)	$\mu_{water}=$ 0.0007	3384	872
Non-Newtonian (Equation (4))	$\mu_{0}=$ 12 $\mu_{\infty }=$ 0.0007	3384	48

**Table 6:** Comparison between mucus discharge between the base and expanded geometry.

Geometry	Viscosity Assumption (Pa·s)	Initial Volume of Mucus or Pus	Volume of Discharged Mucus (pus) After 15 s (mm^3^)	Mucus Discharge Rate
Base geom.	$\mu_{0}=$ 12 $\mu_{\infty }=$ 0.0007	3384	48	1.42%
Extended geom. (×1.05)	$\mu_{0}=$ 12 $\mu_{\infty }=$ 0.0007	3924.2	61.8	1.57%

## Data Availability

The data presented in this study are available on request from the corresponding author. The clinical dataset (CT scans) is not publicly available due to patient privacy and ethical restrictions.
